# ENGAGING PEOPLE WITH DEMENTIA AND THEIR FAMILY IN ADVANCE CARE PLANNING: A 33 COUNTRY DELPHI STUDY

**DOI:** 10.1093/geroni/igad104.1778

**Published:** 2023-12-21

**Authors:** Jenny van der Steen, Ida Korfage

**Affiliations:** Leiden University Medical Center, Leiden, Zuid-Holland, Netherlands; Erasmus MC, University Medical Center Rotterdam, Rotterdam, Zuid-Holland, Netherlands

## Abstract

Reaching a consensus about a definitional framework of advance care planning in dementia was a main goal of an international Delphi study conducted by the European Association for Palliative Care. Four rounds with a panel of 107 experts from 33 countries were conducted between September 2021 and June 2022. The framework comprised a definition and three issues that are of particular importance in the case of dementia, which were capacity, family, and engagement and communication. Focusing on the latter, we provide recommendations that achieved a consensus in the Delphi study. We also interviewed persons with dementia and provide their recommendations for health care providers directly offered by them which the panel found eye-opening. The framework fits dementia and is unique in that it focuses on a communication process that continues independent of the level of capacity, beyond a focus on documenting specific current or end-of-life treatment preferences. It evolved in consensus with experts from western and non-western countries and it should promote inclusiveness of persons with dementia and their family caregivers. The more inclusive conceptualization of what advance care planning in dementia is, provides a much-needed basis for research and policy initiatives on advance care planning in dementia. Further, the work also highlights ambiguity such as about when family caregivers can act as proxies and when not, and the methods may be used as a template for developing frameworks on advance care planning with other specific conditions.

